# Applying Information Gain to Explore Factors Affecting Small-Incision Lenticule Extraction: A Multicenter Retrospective Study

**DOI:** 10.3389/fmed.2022.837092

**Published:** 2022-05-03

**Authors:** Shuang Liang, Shufan Ji, Xiao Liu, Min Chen, Yulin Lei, Jie Hou, Mengdi Li, Haohan Zou, Yusu Peng, Zhixing Ma, Yuanyuan Liu, Vishal Jhanji, Yan Wang

**Affiliations:** ^1^Clinical College of Ophthalmology, Tianjin Medical University, Tianjin, China; ^2^School of Computer Science and Engineering, Beijing University of Aeronautics and Astronautics, Beijing, China; ^3^Qingdao Eye Hospital, Shandong First Medical University, Qingdao, China; ^4^Jinan Mingshui Eye Hospital, Jinan, China; ^5^Department of Health Statistics, College of Public Health, Tianjin Medical University, Tianjin, China; ^6^Department of Ophthalmology, University of Pittsburgh School of Medicine, Pittsburgh, PA, United States; ^7^Tianjin Key Lab of Ophthalmology and Visual Science, Tianjin Eye Hospital, Tianjin Eye Institute, Nankai University Affiliated Eye Hospital, Tianjin, China

**Keywords:** myopia, small-incision lenticule extraction, contributing factors, information gain, multicenter

## Abstract

**Purpose:**

This retrospective study aimed to identify the key factors influencing postoperative refraction after small-incision lenticule extraction (SMILE) using information gain.

**Methods:**

This study comprised 2,350 eyes of 1,200 patients who underwent SMILE using a Visumax 500-kHz femtosecond laser (Carl Zeiss Meditec AG) in three ophthalmic centers: Tianjin Eye Hospital (center A), Jinan Mingshui Eye Hospital (center B), and Qingdao Eye Hospital (center C). Anterior segment features, including corneal curvature and central corneal thickness (CCT), were obtained from Pentacam HR (Oculus, Wetzlar, Germany). Information gain was calculated to analyze the importance of features affecting postoperative refraction.

**Results:**

Preoperative and postoperative mean spherical equivalent (SE) refraction were −5.00 (−6.13, −3.88) D and 0.00 (−0.25, 0.13) D, respectively. None of the patients lost more than two lines of corrected distance visual acuity. The safety index was 1.32 ± 0.24, 1.03 ± 0.08, and 1.13 ± 0.16 in centers A, B, and C, respectively. The efficacy index was 1.31 ± 0.25, 1.02 ± 0.08, and 1.13 ± 0.17 in centers A, B, and C, respectively. At least 95% of the eyes were within ±1.00 D of the attempted correction. Postoperative refraction was related to preoperative spherical diopter refraction (r = 0.369, *p* < 0.001), preoperative SE (r = 0.364, *p* < 0.001), maximum lenticule thickness (r = −0.311, *p* < 0.001), preoperative uncorrected distance visual acuity (r = 0.164, *p* < 0.001), residual stromal thickness (r = 0.139, *p* < 0.001), preoperative mean anterior corneal curvature (r = −0.127, *p* < 0.001), preoperative flattest anterior corneal curvature (r = −0.122, *p* < 0.001), nomogram (r = −0.100, *p* < 0.001) and preoperative CCT (r = −0.058, *p* = 0.005).

**Conclusions:**

SMILE was considered a safe and effective procedure for correcting myopia. Based on information gain, postoperative refraction was influenced by preoperative mean anterior corneal curvature, CCT, refraction, and residual stromal thickness.

## Introduction

Small-incision lenticule extraction (SMILE) is a viable surgical option for the correction of myopia and astigmatism (1). Compared with laser-assisted *in situ* keratomileusis, the SMILE procedure was flapless. Because of no corneal flap, SMILE has the advantages of lower incidence of postoperative dry eye and better stability of corneal biomechanics (2, 3). There is a rising acceptance and recognition of SMILE surgery as a global surgical treatment option for refractive errors (4). Previous studies have reported that sex (5), age (6, 7), preoperative spherical equivalent (SE) (8), corneal curvature (9), optical zone (10), central corneal thickness (CCT) (11, 12), treatment nomogram (13), and laser energy (14, 15) affect visual outcomes after SMILE. While previous studies mostly analyzed the influence of a single factor, in this study, machine learning was used to analyze 20 different factors to determine the most important factors affecting SMILE.

Machine learning has been widely used for the diagnosis of corneal diseases (16), prediction of myopia progression (17), and diagnosis of keratoconus (18). Information gain allows the analysis of the correlation between different variables and their impact on outcomes. The impact of individual features on outcomes can be measured by the information gain (19). Information gain makes a comprehensive consideration of feature selection using the statistical properties of all samples and fitting non-linear data, while multiple linear regression is only capable of analyzing linear data. The purpose of this retrospective study was to explore the factors influencing postoperative refraction after SMILE in different ophthalmic centers using information gain.

## Methods

This retrospective study included patients who underwent SMILE surgery in three ophthalmic centers, namely, Tianjin Eye Hospital (center A), Jinan Mingshui Eye Hospital (center B), and Qingdao Eye Hospital (center C). The inclusion criteria were as follows: age > 18 years, CCT > 450 μm, corrected distance visual acuity (CDVA) of 20/25 or better, stable refraction for the past 2 years and patients demonstrate a keen desire to remove their lenses. Patients stopped wearing soft contact lenses for at least 2 weeks and hard contact lenses for at least 4 weeks before examination. The exclusion criteria were active ocular disease, previous ocular surgery or ocular trauma, keratoconus, psychiatric disorders, and systemic diseases. Informed consent was obtained from all patients. The study protocol was approved by the ethics committee of the Tianjin Eye Hospital (TJYYLL-201914). The study design adhered to the tenets of the Declaration of Helsinki.

### Information Gain

In machine learning applications, information gain is often used for feature selection by evaluating the gain of each feature in the context of the target outcome. The greater the value of the information gain of a feature, the greater the relevance of the feature to the target outcome. The feature with the highest information gain is considered the best feature to be chosen, as it affects the target outcome the most. Information gain can examine the contribution of features to the whole system. It is suitable for the so-called “global” feature selection. In our study, we employed information gain to measure the relevance of some SMILE features, such as residual stromal thickness (RST) and preoperative mean anterior curvature (Pre-Km), to the target SMILE outcome, that is, postoperative SE. The higher the information gain value, the more important the feature is to the SMILE outcome.

Information gain is calculated by the reduction of information entropy, which quantifies the amount of information present in the target outcome.


$$\begin{array}{l} IG\left(S,a\right)\;=\;H\left(S\right)\;-\;H\left(S|a\right) \end{array}$$

where *IG*(*S, a*) is the information gain for the outcome S with feature a, *H*(*S*) is the entropy for the outcome S without feature a, and *H*(*S*|*a*) is the conditional entropy for the outcome S given feature a. The entropy of S can be calculated from the probability distribution p_k, where k can be in K discrete states, and is written as the function *H*(*S*):


$$H\left(S\right)=-\sum_{k}^{K}p_{k}log_{p_{k}}$$

The conditional entropy *H*(*S*|*a*) can be calculated by splitting the dataset into groups for each observed value of a and calculating the sum of the ratio of examples in each group out of the entire dataset multiplied by the entropy of each group, that is,


$$H\left(S|a\right)=\sum_{v}^{a}\frac{Sa\left(v\right)}{S}H\left(Sa\left(v\right)\;\right),$$

where $\frac{Sa\left(v\right)}{S}$ is the ratio of the number of examples in the dataset in which the variable a has the value v, and *H*(*Sa*(*v*)) is the entropy of the group of samples where the variable a has the value v.

In our data analysis, the postoperative SE at 3 months was discretized into three value ranges, 0, 1, and 2, defined as follows: 0:[ −0.25,0.25] D, 1:[ −0.50, −0.25) D or (0.25,0.50] D, and 2: <0.50 D or >0.50 D. Preoperative anterior segment features included flattest anterior corneal curvature (Pre-K1), steepest anterior corneal curvature (Pre-K2), mean anterior corneal curvature (Pre-Km), and preoperative CCT (Pre-CCT). The preoperative features included uncorrected distance visual acuity (UDVA), CDVA, intraocular pressure spherical diopter(Pre-SD), cylinder diopter, cylinder axis, SE, laterality, sex, and age. Surgical design parameters included RST, laser energy, maximum lenticule thickness (Max), cap thickness, optical zone, and treatment nomogram (Nomogram). Information gain values above 0.05 were considered significant.

### Surgical Parameters

The SMILE procedure was performed using a Visumax 500 kHz femtosecond laser (Carl Zeiss Meditec AG) under topical anesthesia in all patients. In centers A, B, and C, the surgical parameters were optical zone 6.2-7.0 mm, cap diameter 7.2-8.0 mm, cap thickness 110-140 μm, and laser energy 125-145 nJ. The SMILE surgery was performed using a standard surgical technique (20) by experienced surgeons at each of the centers.

### Postoperative Treatment and Follow-Up

All patients were prescribed 0.5% levofloxacin (Santen, Inc.) four times a day for 1 week, and 0.1% fluorometholone (Santen, Inc.) four times a day for 1-2 weeks postoperatively. UDVA, CDVA, manifest refraction, and corneal tomography (Pentacam HR, Oculus, Wetzlar, Germany) were performed. The follow-up period is 1 day, 1 week, 1 month, and 3 months after SMILE.

### Statistical Analysis

All analyses were performed using SPSS version 26.0 software (IBM Corp., Armonk, NY, USA) and SAS version 9.4 software (SAS Institute Inc., Cary, NC). The Kolmogorov-Smirnov test was used to test the normality of the data. The data that did not conform to the normal distribution were represented as median (P_25_, P_75_). The relationship between continuous variables, such as Pre-K1, Pre-Km, RST, Max, Pre-CCT, Pre-SD, Pre-SE, Pre-UDVA, Nomogram, and postoperative SE, were analyzed using the Spearman correlation analysis. A effect model was used to analyze the influencing factors. A *p*-value of < 0.05 was regarded as statistically significant.

## Results

**Figure 1:**
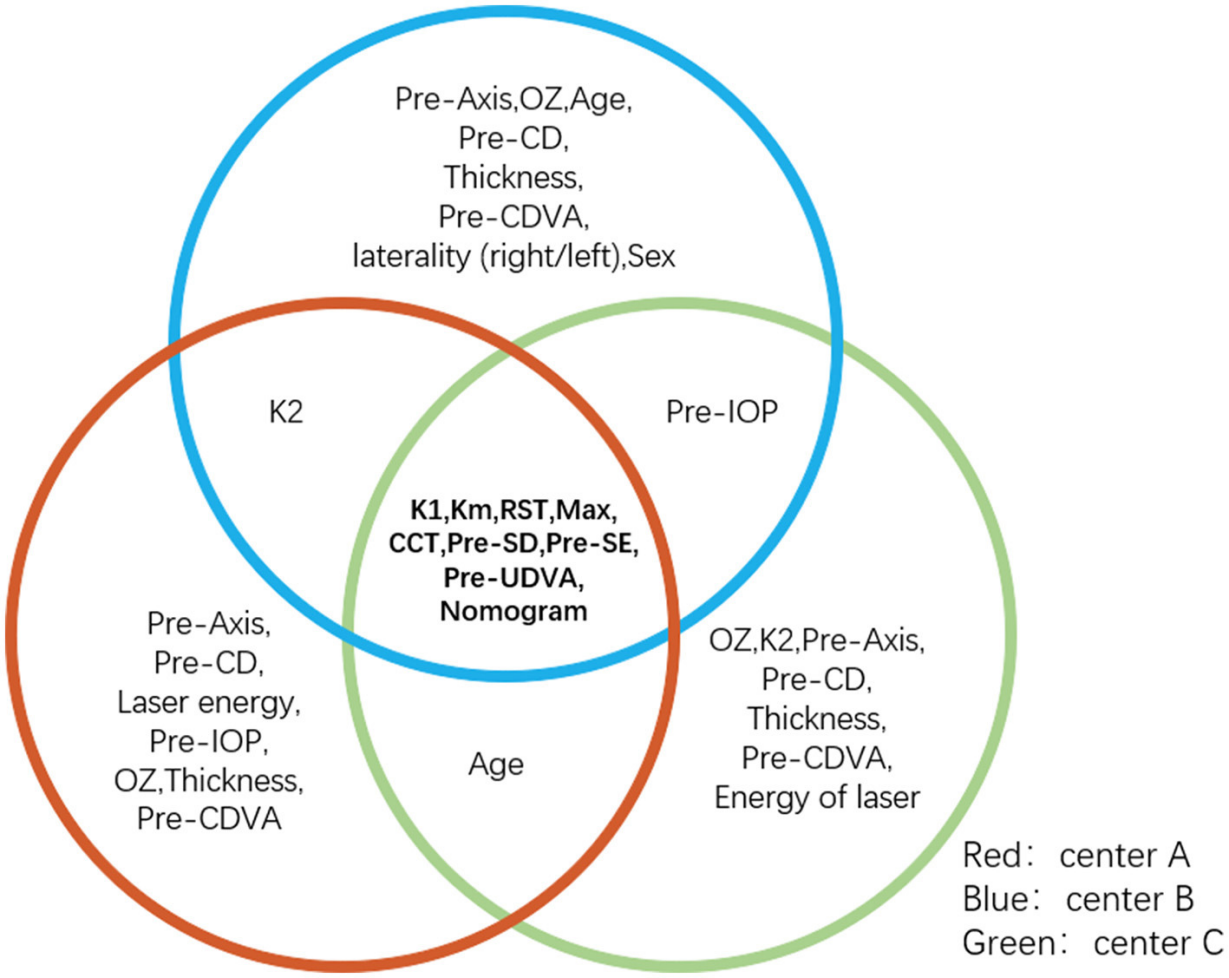
The overlap part of the circle is the feature with three ophthalmic centers information gain values > 0.05, which are considered important factors affecting postoperative SE. K1, Km, RST, Max, CCT, Pre-SD, Pre-SE, Pre-UDVA, and nomogram make a large contribution to postoperative refraction after SMILE. CCT, central corneal thickness; K1, flattest anterior corneal curvature; Km, mean anterior corneal curvature; Max, maximum lenticule thickness; Pre-UDVA, preoperative uncorrected distance visual acuity; Pre-SD, preoperative spherical diopter; Pre-SE, preoperative spherical equivalent; SMILE, small-incision lenticule extraction; RST, residual stromal thickness.

A total of 1,200 subjects (2,350 eyes) were included in this study (60.8% male, 50.6% right eye). The average age of the patients was 20 (18, 21) years. The preoperative SE was −5.00 (−6.13, −3.88) D. Demographic data from the different ophthalmic centers are shown in Table 1. Information gain was used to determine the weight of the factors affecting surgical outcomes. Factors influencing postoperative SE are presented in Table 2. Pre-K1, Pre-Km, RST, Max, Pre-CCT, Pre-SD, Pre-SE, Pre-UDVA, and Nomogram were found to significantly impact postoperative SE in all three centers (Figure 1). The top common nine factors highlighted showed information gain values > 0.05 in all three centers. Other variables, such as thickness, sex, laterality (right/left), and Pre-CDVA, had a smaller effect on postoperative SE.

**Table 1:** Baseline information in the three ophthalmic centers.

	**A**	**B**	**C**	**All**	***P***
Eyes (*N*)	818	702	830	2350	-
Sex (male, %)	51.0	63.4	68.3	60.8	-
Age (years)	21 (9,25)	19 (18,22)	20 (18,23)	20 (18,24)	0.014
Pre-SD (D)	−5.00 (−6.25, −4.00)	−4.38 (−5.75, −3.25)	−4.50 (−5.50, −3.50)	−4.50 (−5.75, −3.50)	0.419
Pre-CD (D)	−0.75 (−1.25, −0.25)	−0.75 (−1.00, −0.25)	−0.50 (−1.00, 0.00)	−0.50 (−1.00, −0.25)	<0.001
Pre-SE (D)	−5.38 (−6.50, −4.38)	−4.75 (−6.13, −3.50)	−4.75 (−5.75, −3.75)	−5.00 (−6.13, −3.88)	0.122
Pre-CCT(μm)	551 (532, 573)	534 (516, 554)	550 (532, 571)	545 (528,568)	<0.001
Pre-Km(D)	43.1 (42.2, 44.0)	43.1 (42.2, 44.1)	42.7 (41.9, 43.6)	43.0 (42.1,43.9)	<0.001

*A, Tianjin Eye Hospital; B, Jinan Mingshui Eye Hospital; C, Qingdao Eye Hospital; N, number of eyes; Pre-SD, preoperative spherical diopter; Pre-CD, preoperative cylinder diopter; Pre-SE, preoperative spherical equivalent. Data are represented as median (P_25_, P_75_). P < 0.05 was regarded as statistically significant*.

**Table 2:** Features affecting postoperative refraction.

**A**		**B**		**C**	
**Feature**	**Information gain value**	** Feature**	**Information gain value**	**Feature**	**Information gain value**
**Pre-K1**	0.0746	**Pre-Km**	0.0831	**Pre-SD**	0.0804
**Pre-SE**	0.0744	**Pre-K2**	0.0801	**OZ**	0.0777
**Pre-Km**	0.0741	**RST**	0.0779	**Nomogram**	0.0696
**Pre-K2**	0.0725	**Pre-SD**	0.0669	**Max**	0.0638
**Age**	0.0721	**Pre-K1**	0.0657	**RST**	0.0619
**RST**	0.0639	**Max**	0.0651	**Pre-SE**	0.0617
**Max**	0.0614	**Pre-CCT**	0.0643	**Pre-CCT**	0.0615
**Pre-SD**	0.0604	**Pre-SE**	0.0606	**Pre-Km**	0.0610
**Pre-CCT**	0.0593	**Pre-UDVA**	0.0536	**Pre-K1**	0.0594
**Nomogram**	0.0574	**Pre-IOP**	0.0527	**Pre-UDVA**	0.0547
**Pre-axis**	0.0535	**Nomogram**	0.05190	**Age**	0.0546
**Pre-CD**	0.0516	Pre-axis	0.0494	**Pre-IOP**	0.0510
**Pre-UDVA**	0.0505	OZ	0.0449	Pre-K2	0.0497
Laser energy	0.0481	Age	0.0446	Pre-axis	0.0495
Pre-IOP	0.0461	Pre-CD	0.0433	Pre-CD	0.0438
OZ	0.0453	Thickness	0.0314	Pre-CDVA	0.0359
Thickness	0.0202	Pre-CDVA	0.0228	Thickness	0.0347
Pre-CDVA	0.0147	Laterality (right/left)	0.0226	Laser energy	0.0291
Sex	0	Sex	0.0193	Sex	0
Laterality (right/left)	0			Laterality (right/left)	0

*Information gain was used to determine the weight of the factors affecting surgical outcomes. The top common nine factors highlighted showed information gain values > 0.05 in all three centers*.

*A, Tianjin Eye Hospital; B, Jinan Mingshui Eye Hospital; C, Qingdao Eye Hospital; Pre-CCT, preoperative central corneal thickness; Pre-K1, preoperative flattest anterior corneal curvature; Pre-K2, preoperative steepest anterior corneal curvature; Pre-Km, preoperative mean anterior corneal curvature; Max, maximum lenticule thickness; OZ, optical zone; Pre-UDVA, preoperative uncorrected distance visual acuity; Pre-CDVA, preoperative corrected distance visual acuity; Pre-IOP, preoperative intraocular pressure; Pre-SD, preoperative spherical diopter; Pre-CD, preoperative cylinder diopter; Pre-axis, preoperative cylinder axis; Pre-SE, preoperative spherical equivalent; RST, residual stromal thickness; Thickness, cap thickness*.

Furthermore, since Table 2 incorporates too many parameters, and each parameter gets a small weight, we repeated the information gain analysis using the nine features found to be significant in Table 2 to obtain a greater weight (Table 3). In Table 3, we selected four out of the top six parameters (shown in bold), whose information gain values were higher than 0.10 in all the three centers. Finally, the result stated that Pre-Km, Pre-CCT, Pre-SD, and Pre-SE were the most influential features affecting postoperative refraction in all the three centers.

**Table 3:** Secondary information gain of the nine most influential feature (highlighted in Table 2) affecting postoperative refraction.

**A**		**B**		**C**	
**Feature**	**Information gain value**	** Feature**	**Information gain value**	**Feature**	**Information gain value**
**Pre-Km**	0.1761	**Pre-Km**	0.1584	Nomogram	0.1360
Max	0.1425	Pre-K1	0.1241	**Pre-SE**	0.1326
**Pre-CCT**	0.1209	**Pre-CCT**	0.1190	Max	0.1249
Pre-K1	0.1167	**Pre-SD**	0.1127	**Pre-SD**	0.1215
**Pre-SD**	0.1093	Pre-RST	0.1118	**Pre-Km**	0.1056
**Pre-SE**	0.1058	**Pre-SE**	0.1069	**Pre-CCT**	0.1001
Pre-UDVA	0.0959	Max	0.1047	Pre-K1	0.0992
Nomogram	0.0678	Pre-UDVA	0.0878	RST	0.0966
RST	0.0652	Nomogram	0.0747	Pre-UDVA	0.0836

*Information gain analysis again using the top nine parameters (highlighted in Table 2) obtained above. The features that showed high information gain values (>0.10) in all three centers are highlighted. The four top-ranking parameters (shown in bold) were selected as the most important factors affecting SMILE surgery*.

*A, Tianjin Eye Hospital; B, Jinan Mingshui Eye Hospital; C, Qingdao Eye Hospital; Pre-CCT, preoperative central corneal thickness; Pre-K1, preoperative flattest anterior corneal curvature; Pre-Km, preoperative mean anterior corneal curvature; Max, maximum lenticule thickness; Pre -UDVA, preoperative uncorrected distance visual acuity; Pre-SD, preoperative spherical diopter; Pre-SE, preoperative spherical equivalent; RST, residual stromal thickness*.

The result of the correlation analysis of the patients in all the three centers is displayed in Table 4. Postoperative SE was related to Pre-SD (r = 0.369, *p* < 0.001), Pre-SE (r = 0.364, *p* < 0.001), Max (r = −0.311, *p* < 0.001), Pre-UDVA (r = 0.164, *p* < 0.001), RST (r = 0.139, *p* < 0.001), Pre-Km (r = −0.127, *p* < 0.001), Pre-K1 (r = −0.122, *p* < 0.001), nomogram (r = −0.100, *p* < 0.001), and Pre-CCT (r = −0.058, *p* = 0.005).

**Table 4:** The result of the correlation analysis.

	**Pre-K1**	**Pre-Km**	**RST**	**Max**	**Pre-CCT**	**Pre-SD**	**Pre-SE**	**Pre-UDVA**	**Nomogram**
R	−0.122	−0.127	0.139	−0.311	−0.058	0.369	0.364	0.164	−0.100
Correlation	Negative	Negative	Positive	Negative	Negative	Positive	Positive	Positive	Negative
*p*	<0.001	<0.001	<0.001	<0.001	0.005	<0.001	<0.001	<0.001	<0.001

*Spearman was applied to analyze the correlation of factors affecting the postoperative spherical equivalent in all three centers. P < 0.05 was regarded as statistically significant*.

*Pre-K1, preoperative flattest anterior corneal curvature; Pre-Km, preoperative mean anterior corneal curvature; RST, residual stromal thickness; Max, maximum lenticule thickness; Pre-CCT, preoperative central corneal thickness; Pre-SD, preoperative spherical diopter; Pre-SE, preoperative spherical equivalent; Pre-UDVA, preoperative uncorrected distance visual acuity*.

The results of random effects estimation for the null model is shown in Table 5. The null model is the first step for building mixed effect model and is used to determine whether the construction of the mixed effect model is necessary. The results of null model indicate that the correlation in laterality is not statistically significant (p1 = 0.143, p2 = 0.106). It means that there is no significant difference between right eye, left eye and binocular.

**Table 5:** The results of random effects estimation in laterality for the null model.

**Cov Parm**	**Subject**	**Group**	**Estimate**	**Standard error**	***t***	***P***
CHOL(1,1)	laterality	Group 0	0.4313	0.2974	4.38	0.143
CHOL(1,1)		Group 1	0		5.96	0.106
CHOL(1,1)		Group 2	0.5			

*The null model was applied for random effects estimation in laterality*.

*Laterality represents the operation eye (Group 0, right eye; Group 1, left eye; Group 2, both eyes). P < 0.05 was regarded as statistically significant*.

### Standard Refractive Analyses

**Figure 2:**
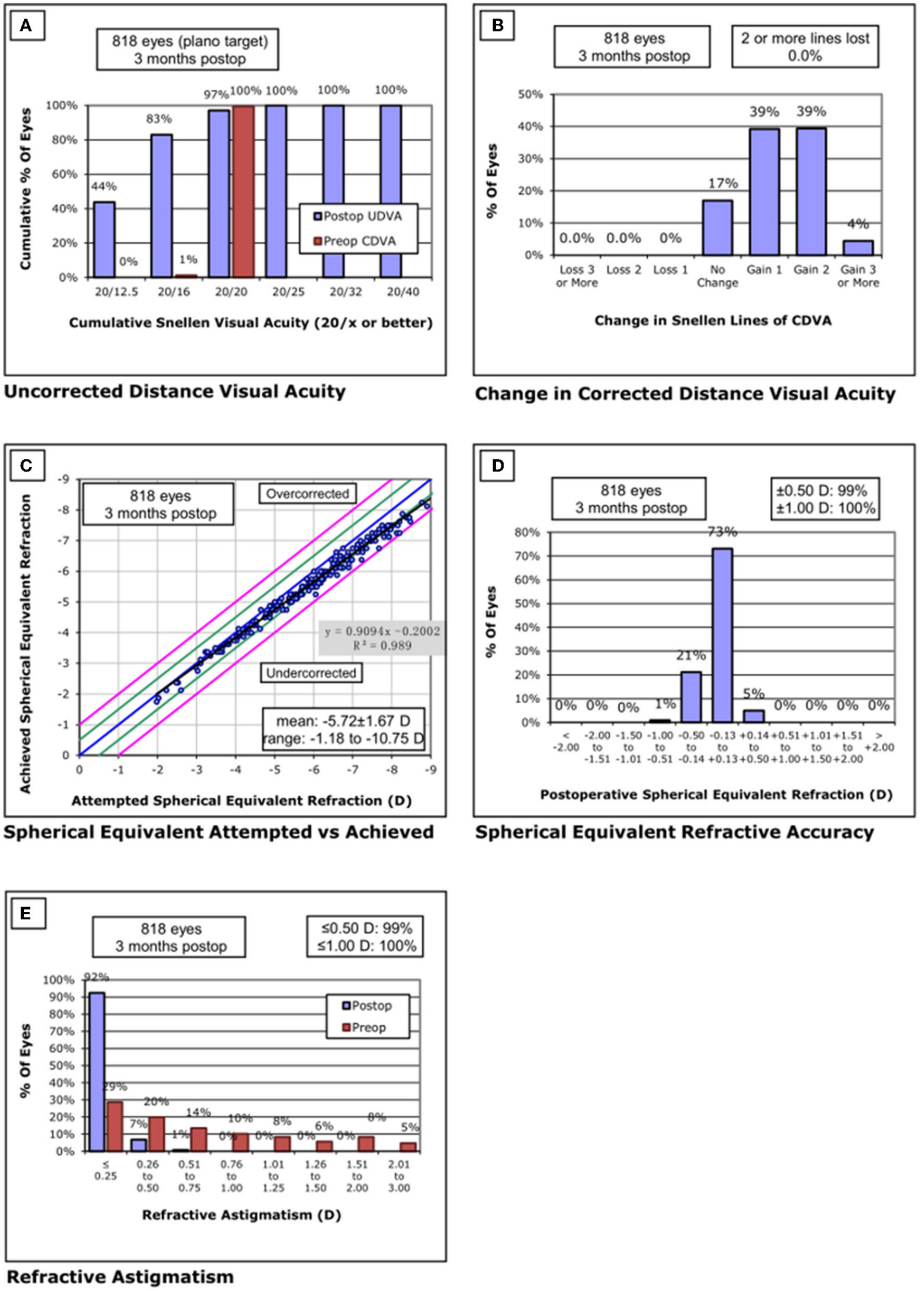
Standard graphs of refractive surgery visual and refractive outcomes for 830 eyes at 3 months post-SMILE in center A. **(A)** Uncorrected distance visual acuity. **(B)** Change in corrected distance visual acuity. **(C)** Spherical equivalent attempted vs. achieved. **(D)** Spherical equivalent refractive accuracy. **(E)** Refractive astigmatism. SMILE, small-incision lenticule extraction.

**Figure 3:**
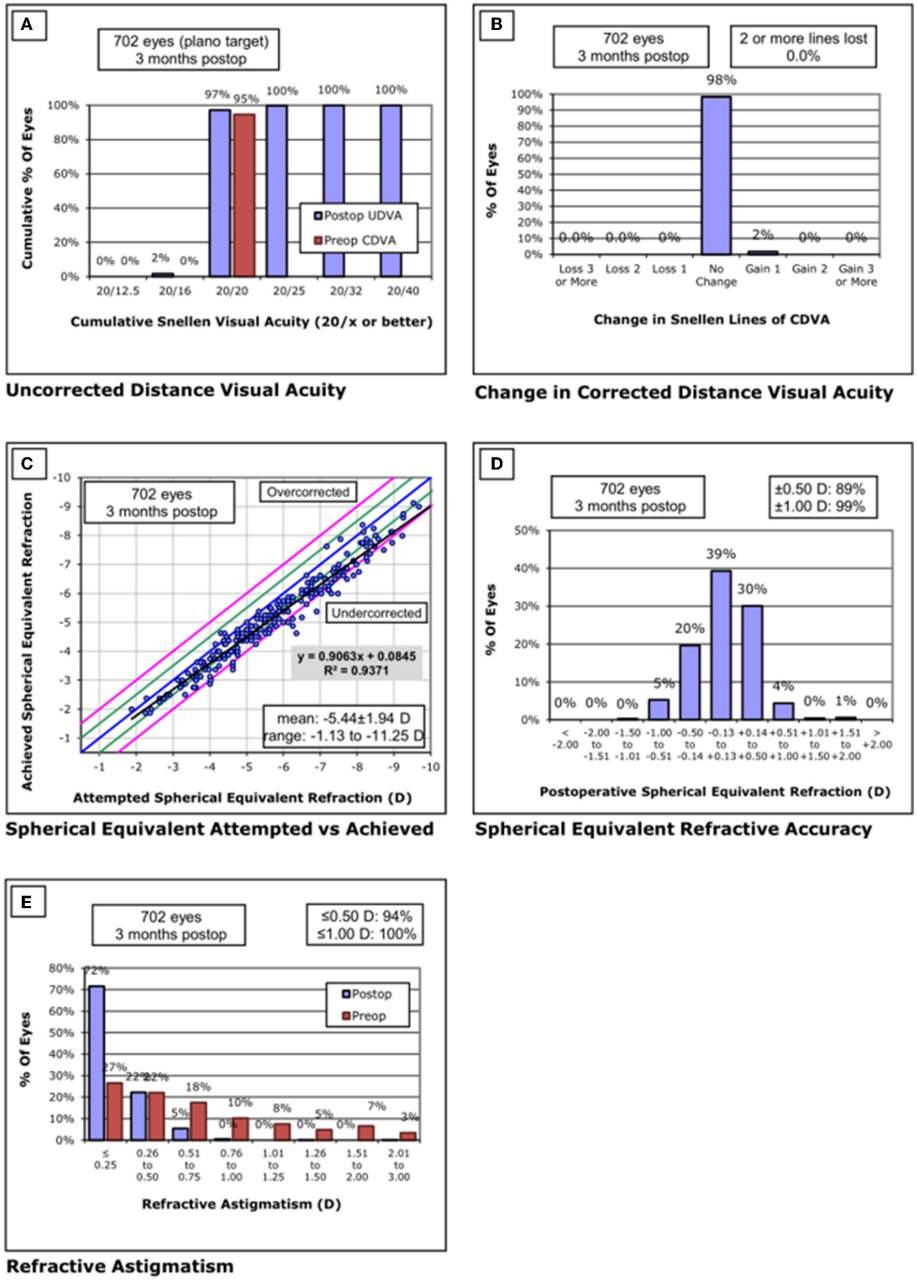
Standard graphs of refractive surgery visual and refractive outcomes for 702 eyes at 3 months post-SMILE in center B. **(A)** Uncorrected distance visual acuity. **(B)** Change in corrected distance visual acuity. **(C)** Spherical equivalent attempted vs. achieved. **(D)** Spherical equivalent refractive accuracy. **(E)** Refractive astigmatism. SMILE, small-incision lenticule extraction.

**Figure 4:**
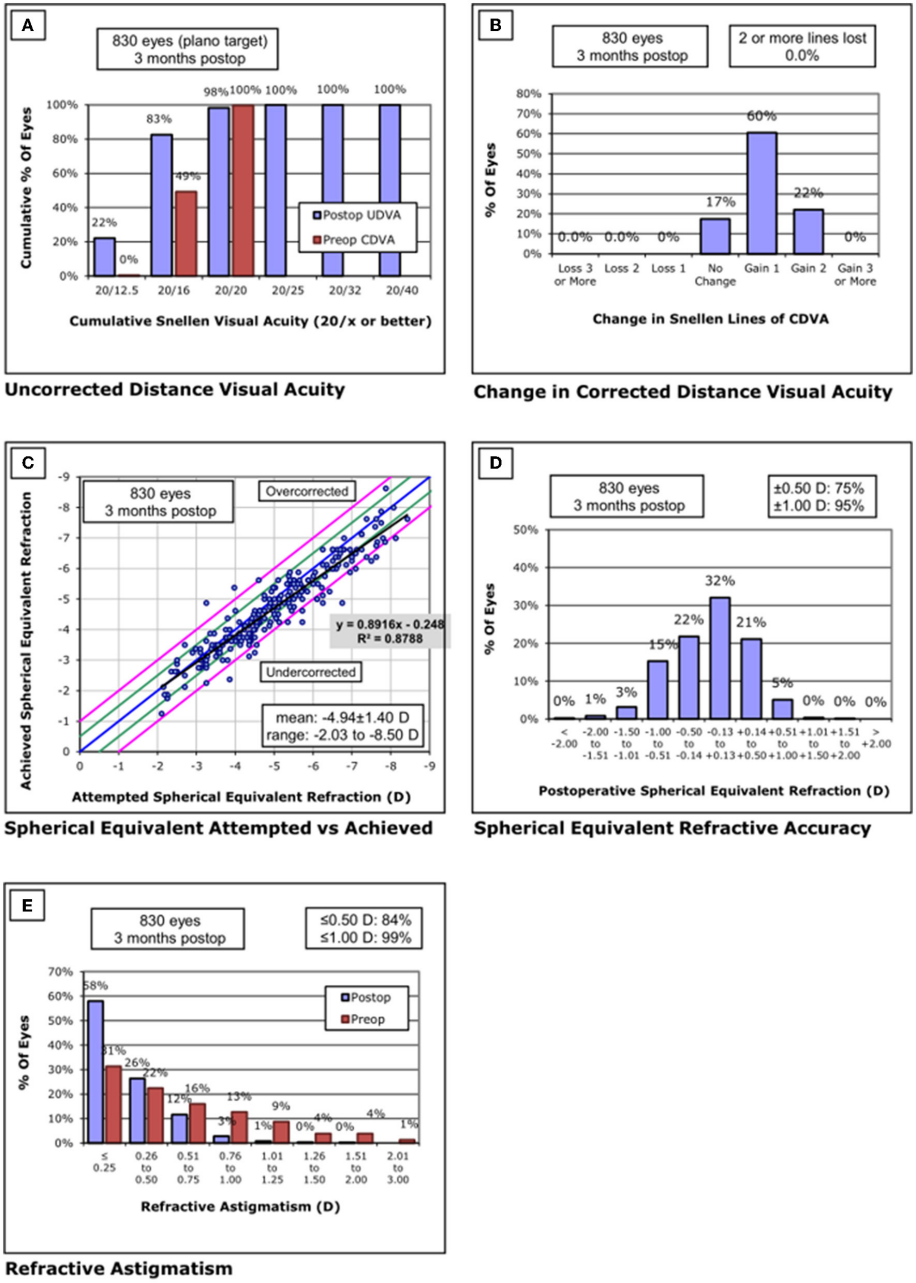
Standard graphs of refractive surgery visual and refractive outcomes for 830 eyes at 3 months post-SMILE in center C. **(A)** Uncorrected distance visual acuity. **(B)** Change in corrected distance visual acuity. **(C)** Spherical equivalent attempted vs. achieved. **(D)** Spherical equivalent refractive accuracy. **(E)** Refractive astigmatism. SMILE, small-incision lenticule extraction.

Standardized graphs of surgical outcomes after SMILE are displayed in Figures 2–4. There was no intraoperative or postoperative complications in all centers.

#### Center A

None of the eyes lost two or more lines of CDVA. The safety index was 1.32 ± 0.24. Throughout the follow-up, the UDVA was 20/20 or better in 794/818 eyes (97%) and equal to or better than the preoperative CDVA in 818/818 eyes (100%). The efficacy index was 1.31 ± 0.25. The postoperative SE was within ±0.50 D of the attempted correction in 99% of eyes and within ±1.00 D in all the eyes.

#### Center B

None of the eyes lost two or more lines of CDVA. The safety index was 1.03 ± 0.08. Throughout the follow-up, the UDVA was 20/20 or better in 682/702 eyes (97%) and equal to or better than the preoperative CDVA in 702/702 eyes (100%). The efficacy index was 1.02 ± 0.08. Postoperative SE was within ±0.50 D of the attempted correction in 89% of the eyes and within ±1.00D in 99% of the eyes.

#### Center C

None of the eyes lost two or more lines of CDVA. The safety index was 1.13 ± 0.16. Throughout the follow-up, the UDVA was 20/20 or better in 816/830 eyes (98%) and equal to or better than the preoperative CDVA in 830/830 eyes (100%). The efficacy index was 1.13 ± 0.17. Postoperative SE was within ±0.50 D of the attempted correction in 75% of the eyes and within ±1.00D in 95% of the eyes.

## Discussion

The safety, efficacy, and predictability of SMILE were confirmed in all the patients in our study. After analyzing a total of 20 parameters, including anterior segment features, preoperative parameters, and surgical design parameters, valuable and interesting results were obtained. Corneal curvature, CCT, SD, SE, UDVA, RST, maximum lenticule thickness, and nomogram were the factors affecting postoperative refraction after SMILE. In addition, mean anterior corneal curvature, CCT, SD, and SE were the most influential features of postoperative refraction among the nine common features.

There are various factors that impact the SMILE procedure in order to obtain better vision outcome. In the study, the contribution of each parameter was obtained by combining the data in multicenter, so that the top factors influencing the surgery were acquired. The factors that influence the postoperative refraction of SMILE include not only corneal parameters but also preoperative refraction and surgical parameters. Our findings indicate that preoperative corneal parameters, including Pre-Km (r = −0.127, *p* < 0.001), Pre-K1 (r = −0.122, *p* < 0.001), and Pre-CCT(r = −0.058, *p* = 0.005), play a crucial role in postoperative refraction after SMILE. The diverse ocular biometric parameters are interactive. This result is consistent with that of a previous study in which in the eyes with low myopia, a steeper corneal curvature could lead to a greater undercorrections after SMILE (9), suggesting that a steeper corneal curvature is often associated with high myopia, which tends to be undercorrected after SMILE (22, 23). In the current study, the entire corneal thickness was negatively correlated with the postoperative SE. This might be attributed to the differences in corneal biomechanics based on corneal thickness (24).

Our study showed that preoperative refraction parameters, including Pre-SD (r = 0.369, *p* < 0.001), Pre-SE (r = 0.364, *p* < 0.001), and Pre-UDVA (r = 0.164, *p* < 0.001), had a positive correlation with postoperative SE after SMILE. A higher preoperative SD or SE is associated with a greater postoperative SE after photorefractive keratectomy, laser-assisted *in situ* keratomileusis, or SMILE (21, 25, 26). In addition, in our study, the higher the preoperative UDVA, the greater the postoperative SE, demonstrating that preoperative UDVA is somewhat predictive of postoperative surgical outcomes. Cui et al. (13) indicated that preoperative UDVA can affect the nomogram in SMILE, which may explain why preoperative UDVA plays a role in postoperative SE. Much more attention should be paid to the patient's preoperative UDVA in future studies to improve surgical outcomes.

Among surgical parameters, RST (r = 0.139, *p* < 0.001), Max (r = −0.311, *p* < 0.001), and nomogram (r = −0.100, *p* < 0.001) were noted to influence postoperative SE after SMILE. Ogasawara et al. (27) suggested that RST correlated with regression of myopia after laser-assisted *in situ* keratomileusis during long-term follow-up and that adequate RST is important to preserve a good UDVA. Nevertheless, there was no obvious correlation between UDVA and postoperative SE. In this study, preserving more RST was beneficial in obtaining a greater postoperative SE. It is worth noting that the maximum lenticule thickness represents the actual corneal ablation depth. A tendency for undercorrection after surgery for high myopia compared to mild to moderate myopia is well documented (21). Evidence indicates that the nomogram plays an important role in the safety, efficacy, and predictability of corneal refractive surgery (28). In the eyes with high myopia 1 year after SMILE, the SE was significantly worse. Adjustment of the nomogram to 0.13 × attempted SE (D)-0.66 D has been suggested (23). In summary, more degrees need to be added in high myopia for correction.

Corneal cap thickness, sex, laterality (right/left), laser energy, and preoperative CDVA did not affect postoperative refraction in our cohort. Liu et al. (12) have demonstrated that a 110-μm cap thickness had better visual outcomes postoperatively compared with a 150-μm cap thickness. In contrast, another study found that postoperative refraction was not significantly affected by cap thickness of 100 and 120 μm in SMILE (11). In our study, cap thickness ranged from 110 to 140 μm, which may have led to different results. In contrast with a previous study on the impact of the energy setting on visual outcomes after SMILE (14), the influence of laser energy was clinically insignificant in our study. Although a Visumax 500 kHz femtosecond laser was used in all patients, the temperature or humidity settings might have been different in the three centers. In addition, the large number of parameters analyzed might explain why laser energy contributed less to postoperative refraction.

The current study has both strengths and limitations. Due to the strong covariance of the data, the linear model is not effective at the beginning of this study. However, Applying information gain, a ranking of the importance of 20 features affecting postoperative SE was derived in the study. In particular, although its design was retrospective, this study included a large number of eyes from three ophthalmic centers. However, different surgical setups may result in measurement errors. In addition, further statistical analysis in the study revealed that no correlation was found between monocular and binocular, which may reduce the possible risk of wrong results due to the violation of the assumption of independence. Finally, the outcomes in center A varied widely compared to those of centers B and C. The reason for this is that the surgeon in center A has extensive experience and has been performed more than 10,000 SMILE procedures since 2011.

In summary, our study assessed factors affecting postoperative refraction after SMILE. Among 20 parameters evaluated in three ophthalmic centers, preoperative mean anterior corneal curvature, CCT, SD, and SE significantly affected postoperative refraction. A larger preoperative Km and CCT is associated with a smaller preoperative SD and RST and a smaller postoperative SE. These findings can be used to optimize the outcomes of SMILE surgery. The refractive surgery surgeons should pay more attention to the patient's preoperative Km, CCT, SD and RST in the daily routine to obtain great outcome for postoperative refraction.

## Data Availability Statement

The original contributions presented in the study are included in the article/supplementary materials, further inquiries can be directed to the corresponding author/s.

## Ethics Statement

The studies involving human participants were reviewed and approved by Ethical Committee of Tianjin Eye Hospital. The patients/participants provided their written informed consent to participate in this study.

## Author Contributions

YW, SL, SJ, MC, and YLe designed and performed the research. SL and XL organized the manuscript writing. XL, SJ, and YLi analyzed the data. JH, ML, HZ, YP, and ZM collected the data. VJ and YW reviewed the manuscript. YW obtained funding. All authors contributed to the article and approved the submitted version.

## Funding

This study was supported by the National Natural Science Foundation of China (No. 81873684). The funding organization had no role in the design or conduct of this research.

## Conflict of Interest

The authors declare that the research was conducted in the absence of any commercial or financial relationships that could be construed as a potential conflict of interest.

## Publisher's Note

All claims expressed in this article are solely those of the authors and do not necessarily represent those of their affiliated organizations, or those of the publisher, the editors and the reviewers. Any product that may be evaluated in this article, or claim that may be made by its manufacturer, is not guaranteed or endorsed by the publisher.

## Abbreviations

SMILE: small-incision lenticule extraction
CCT: central corneal thickness
SE: spherical equivalent
CDVA: corrected distance visual acuity
UDVA: uncorrected distance visual acuity
K1: flattest anterior corneal curvature
Km: mean anterior corneal curvature
Max: maximum lenticule thickness
Pre-UDVA: preoperative uncorrected distance visual acuity
Pre-SD: preoperative spherical diopter
Pre-SE: preoperative spherical equivalent
Pre-Km: preoperative mean anterior curvature
Pre-K1: preoperative flattest anterior corneal curvature
Pre-K2, preoperative steepest anterior corneal curvature, RST: residual stromal thickness.
